# Supplementary material for: Increased mobilization of mesenchymal stem cells in patients with acute respiratory distress syndrome undergoing extracorporeal membrane oxygenation
Source: PLoS One. 2020 Jan 27;15(1):e0227460. doi: 10.1371/journal.pone.0227460 (PMC6984734; doi:10.1371/journal.pone.0227460)
Supplement: S1 Fig — (DOCX) [file pone.0227460.s001.docx]

**Supplemental Figure 1.**


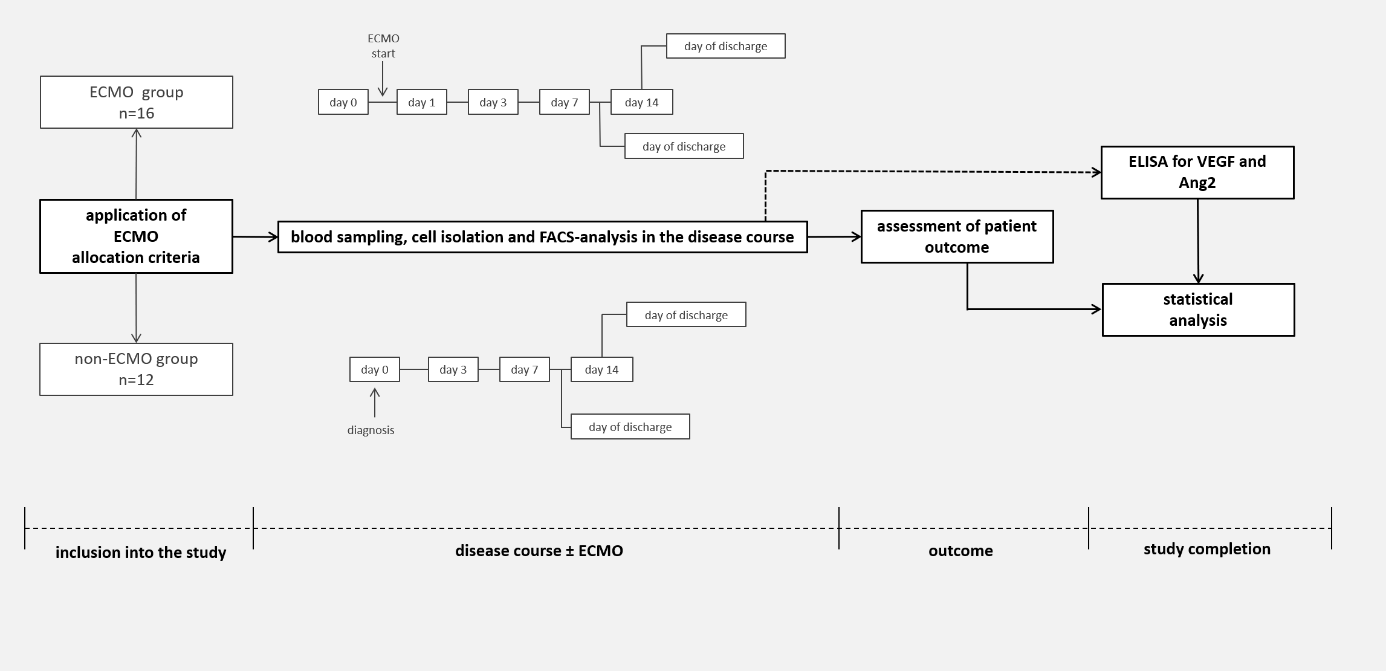


**Supplemental Figure 1: Timeline-Flowchart depicting timepoints of blood sampling.**
