# Supplementary material for: Increased mobilization of mesenchymal stem cells in patients with acute respiratory distress syndrome undergoing extracorporeal membrane oxygenation
Source: PLoS One. 2020 Jan 27;15(1):e0227460. doi: 10.1371/journal.pone.0227460 (PMC6984734; doi:10.1371/journal.pone.0227460)
Supplement: S2 Fig — Numbers of endothelial progenitor cell (EPC) and mesenchymal stem cell (MSC) subpopulations (panel A) as well as serum levels of vascular endothelial growth factor (VEGF) (panel B) and angiopoietin 2 (Ang2) (panel C) are shown for the survivors and non-survivors in the ECMO-dependent and the ECMO-independent group. *marks a significant difference (p<0.05). (DOCX) [file pone.0227460.s002.docx]

**Supplemental Figure 2.**


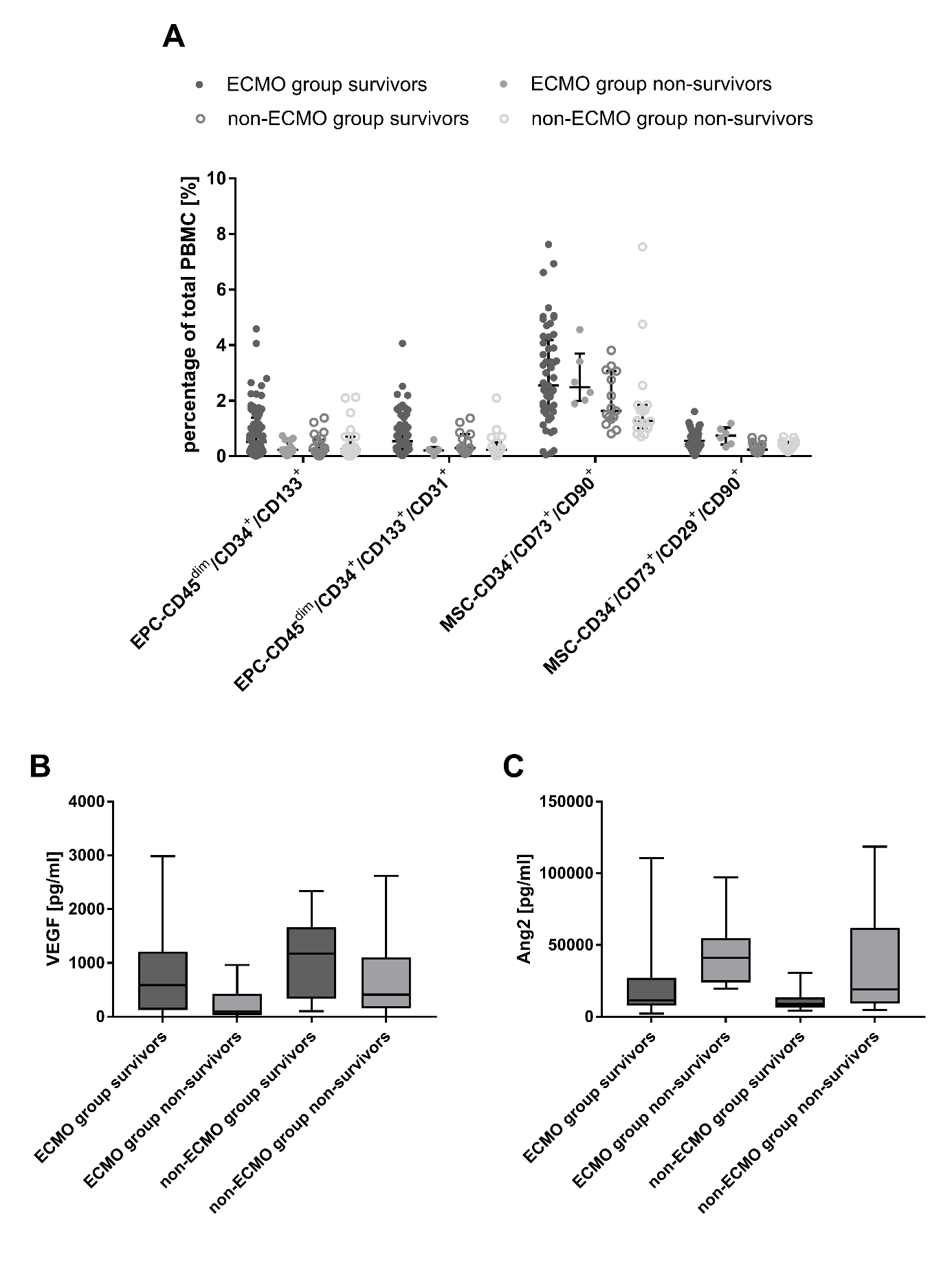


**Supplemental Figure 2.** **Association of survival with numbers of EPC and MSC as well as mobilizing factors in the ECMO-dependent and the ECMO-independent group.** Numbers of endothelial progenitor cell (EPC) and mesenchymal stem cell (MSC) subpopulations (panel A) as well as serum levels of vascular endothelial growth factor (VEGF) (panel B) and angiopoietin 2 (Ang2) (panel C) are shown for the survivors and non-survivors in the ECMO-dependent and the ECMO-independent group.
