# Supplementary material for: Increased mobilization of mesenchymal stem cells in patients with acute respiratory distress syndrome undergoing extracorporeal membrane oxygenation
Source: PLoS One. 2020 Jan 27;15(1):e0227460. doi: 10.1371/journal.pone.0227460 (PMC6984734; doi:10.1371/journal.pone.0227460)
Supplement: S1 Table — (DOCX) [file pone.0227460.s003.docx]

**Supplementary Table 1**

**Supplementary Table 1** shows Pearson correlation coefficients between EPC subpopulations and VEGF serum levels at day 0 and in the disease course.

|  | **day 0** | | **disease course** | |
| --- | --- | --- | --- | --- |
| **EPC subpopulation** | Corr.coef. | p-value | Corr.coef. | p-value |
| CD45^dim^/CD34^+^/CD133^+^ | 0.55 | 0.01 | 0.37 | 0.02 |
| CD45^dim^/CD34^+^/CD133^+^/CD31^+^ | 0.61 | 0.01 | 0.45 | 0.01 |

*EPC*, endothelial progenitor cells; *CD*, cluster of differentiation.
