# Supplementary material for: Increased mobilization of mesenchymal stem cells in patients with acute respiratory distress syndrome undergoing extracorporeal membrane oxygenation
Source: PLoS One. 2020 Jan 27;15(1):e0227460. doi: 10.1371/journal.pone.0227460 (PMC6984734; doi:10.1371/journal.pone.0227460)
Supplement: S2 Table — (DOCX) [file pone.0227460.s004.docx]

**Supplementary Table 2**

**Characteristics and laboratory findings in ARDS patients at day 0**

|  | **ECMO group** | **non-ECMO group** |  |
| --- | --- | --- | --- |
|  | **mean** | **mean** | p |
|  |  |  |  |
| SAPS2 | 52,9 ± 17,9 | 46,4 ± 13,6 | 0,27 |
| TISS | 24,4 ± 8,13 | 24,3 ± 13,2 | 0,53 |
| c-reactive protein [mg/l] | 202 ± 122 | 288 ± 147 | 0,28 |
| procalcitonin [µg/l] | 18,6 ± 17,2 | 5,3 ± 7,83 | **0,02** |

Values for SAPS2, TISS, c-reactive protein and procalcitonin are shown as mean ± SD.

*ND*, no data available; *SAPS II*, Simplified Acute Physiology Score II; *TISS*, Therapeutic Intervention Scoring System (TISS)
